# Translatome and transcriptome analysis of TMA20 (MCT-1) and TMA64 (eIF2D) knockout yeast strains

**DOI:** 10.1016/j.dib.2019.103701

**Published:** 2019-02-02

**Authors:** Desislava S. Makeeva, Andrey S. Lando, Aleksandra Anisimova, Artyom A. Egorov, Maria D. Logacheva, Alexey A. Penin, Dmitry E. Andreev, Pavel G. Sinitcyn, Ilya M. Terenin, Ivan N. Shatsky, Ivan V. Kulakovskiy, Sergey E. Dmitriev

**Affiliations:** a Belozersky Institute of Physico-Chemical Biology, Lomonosov Moscow State University, Moscow, 119234 Russia; b School of Bioengineering and Bioinformatics, Lomonosov Moscow State University, Moscow, 119234 Russia; c Vavilov Institute of General Genetics, Russian Academy of Sciences, Moscow, 119991 Russia; d Department of Medical Physics, Faculty of Physics, Lomonosov Moscow State University, Moscow, 119991 Russia; e Skolkovo Institute of Science and Technology, Moscow, 121205 Russia; f Department of Genetics, Faculty of Biology, Lomonosov Moscow State University, Moscow, 119991 Russia; g Institute for Information Transmission Problems, Russian Academy of Sciences, Moscow, Russia; h Sechenov First Moscow State Medical University, Institute of Molecular Medicine, Moscow, 119991 Russia; i Institute of Mathematical Problems of Biology RAS - the Branch of Keldysh Institute of Applied Mathematics of Russian Academy of Sciences, Pushchino, Moscow Region, 142290 Russia; j Engelhardt Institute of Molecular Biology, Russian Academy of Sciences, Moscow 119991, Russia; k Department of Biochemistry, Biological Faculty, Lomonosov Moscow State University, Moscow, 119234 Russia

## Abstract

TMA20 (MCT-1), TMA22 (DENR) and TMA64 (eIF2D) are eukaryotic translation factors involved in ribosome recycling and re-initiation. They operate with P-site bound tRNA in post-termination or (re-)initiation translation complexes, thus participating in the removal of 40S ribosomal subunit from mRNA stop codons after termination and controlling translation re-initiation on mRNAs with upstream open reading frames (uORFs), as well as *de novo* initiation on some specific mRNAs. Here we report ribosomal profiling data of *S.cerevisiae* strains with individual deletions of *TMA20*, *TMA64* or both *TMA20* and *TMA64* genes. We provide RNA-Seq and Ribo-Seq data from yeast strains grown in the rich YPD or minimal SD medium. We illustrate our data by plotting differential distribution of ribosomal-bound mRNA fragments throughout uORFs in 5′-untranslated region (5′ UTR) of GCN4 mRNA and on mRNA transcripts encoded in MAT locus in the mutant and wild-type strains, thus providing a basis for investigation of the role of these factors in the stress response, mating and sporulation. We also document a shift of transcription start site of the *APC4* gene which occurs when the neighboring *TMA64* gene is replaced by the standard G418-resistance cassette used for the creation of the Yeast Deletion Library. This shift results in dramatic deregulation of the *APC4* gene expression, as revealed by our Ribo-Seq data, which can be probably used to explain strong genetic interactions of *TMA64* with genes involved in the cell cycle and mitotic checkpoints. Raw RNA-Seq and Ribo-Seq data as well as all gene counts are available in NCBI Gene Expression Omnibus (GEO) repository under GEO accession GSE122039 (https://www.ncbi.nlm.nih.gov/geo/query/acc.cgi?acc=GSE122039).

**Specifications table**

**Table:** 

Subject area	Biology
More specific subject area	Protein Synthesis, Translational Control, Ribosome, Bioinformatics, Transcriptomics, Translatomics
Type of data	Table and figures
How data was acquired	Ribosome profiling and RNA-Seq of wild-type or knockout yeast strains were performed. Sequences were obtained using Illumina HiSeq. 2000.
Data format	Raw and analyzed
Experimental factors	*Saccharomyces cerevisiae* BY4741 wild-type strain and BY4741-based strains with TMA20, TMA64 or both TMA20 and TMA64 knockouts were maintained in rich (YPD) or minimal (SD) media.
Experimental features	In the mid-log exponential phase, yeast cells were pretreated with cycloheximide and collected. cDNA libraries of ribosome-bound mRNA and total mRNA from wild-type and knockout strains were performed as described previously [1]. Sequenced reads were trimmed, read mapping and counting was performed.
Data source location	Moscow State University (Moscow, Russia)
Data accessibility	Analyzed data is presented in the article. Raw RNA-Seq and Ribo-Seq data as well as all gene counts are available in NCBI Gene Expression Omnibus (GEO) repository under GEO accession GSE122039 (https://www.ncbi.nlm.nih.gov/geo/query/acc.cgi?acc=GSE122039).
Related research article	N/A

**Value of the data**

• The data provides a gene expression landscape of yeast strains lacking TMA20 and/or TMA64 proteins, which are orthologous to mammalian translation factors MCT-1 and eIF2D, thus expanding our knowledge about individual functional roles of these two translation factors in a living cell.
• An abnormal translation of the MATa2 mRNA derived from the MAT locus of MATa yeast strain is detected, which can be used for explanation of sporulation defects previously detected in the TMA64 deletion strain.
• Quantitative Ribo-Seq data provides essential information of translational changes in the knockout strains including altered uORFs translation in 5’ UTR of mRNA encoding important transcription regulator GCN4, thus providing a basis for investigating the role of these proteins in the stress response.
• The RNA-Seq data highlights transcription abnormalities within the *APC4* gene locus, caused by replacement of the adjacent *TMA64* gene by the standard G418 or HYG resistance cassettes commonly used for generating gene deletions, which can be probably used to explain previously observed strong genetic interactions of *TMA64* with genes involved in the cell cycle and mitotic checkpoints.
• The deep sequenced Ribo-Seq and RNA-Seq are applicable for detailed bioinformatics analysis of translation events, such as prediction of alternative open reading frames.

## 1 Data

**Fig. 1:**
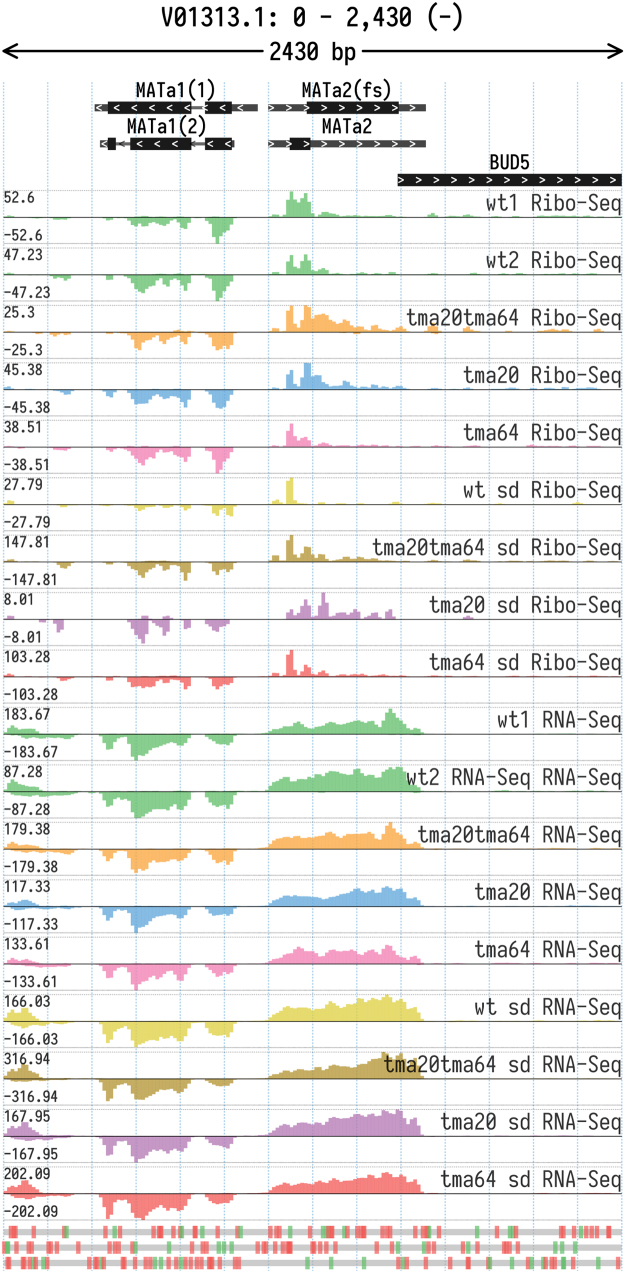
Ribo-Seq and RNA-Seq coverage of MATa locus in the studied yeast strains. According to Ribo-Seq signals, MATa2-2 ORF is probably translated in mutant strains. The Y axis tracks show total read coverage (positive and negative values correspond to the coverage of the direct and reverse complementary strands respectively).

**Fig. 2:**
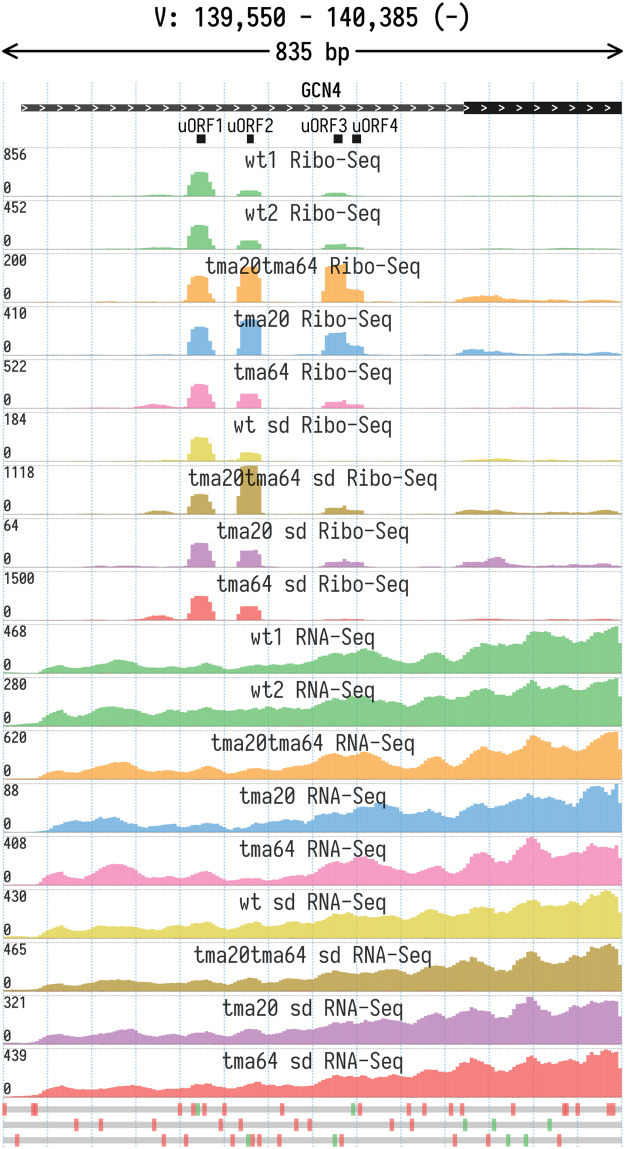
Ribo-Seq and RNA-Seq coverage of the 5′ UTR of the yeast GCN4 gene. Ribo-Seq profiles show ribosome occupancy at different uORFs that regulate translation of the main coding region. The Y axis tracks show total read coverage (positive and negative values correspond to the coverage of the forward and reverse complementary strands respectively).

**Fig. 3:**
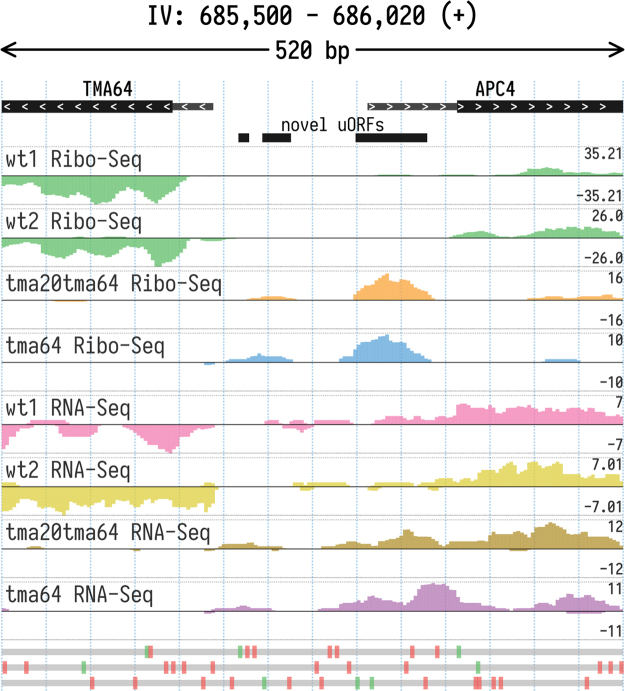
Ribo-Seq and RNA-Seq coverage of the region between the *TMA64* and *APC4* genes. Data suggests that strains with *TMA64* knockout exhibit extended 5′ UTR of APC4 gene leading to translation of novel uORFs. The Y axis tracks show total read coverage (positive and negative values correspond to the coverage of the forward and reverse complementary strands respectively).

In this study we present ribosome profiling data generated from the wild-type BY4741 *S.cerevisiae* strain and strains lacking translation factors TMA20 (MCT-1), TMA64 (eIF2D) or both of them at the same time. Information on all performed experiments is shown in Table 1. Raw Ribo-Seq and RNA-Seq data are available online in the NCBI Gene Expression Omnibus repository (GEO accession: GSE122039, https://www.ncbi.nlm.nih.gov/geo/query/acc.cgi?acc=GSE122039). Supplementary Table S1 and Supplementary Figure S1 contain analyzed NGS data, as described below. Examples of differentially translated and transcribed genes in wild-type and knockout strains are presented in Fig. 1, Fig. 2, Fig. 3.

**Table 1:** Summary of datasets obtained in the study.

Sample	Sample name	Yeast strain name	Yeast strain genotype	Growth media	Sample type
1	wt1_ribo	wt	*MATa his3Δ1 leu2Δ0 met15Δ0 ura3Δ0*	YPD	Ribo-Seq
2	wt1_rna			YPD	RNA-Seq
3	wt2_ribo			YPD	Ribo-Seq
4	wt2_rna			YPD	RNA-Seq
5	wt_sd_ribo			SD	Ribo-Seq
6	wt_sd_rna			SD	RNA-Seq
7	tma20_ribo	Δtma20	*MATa his3Δ1 leu2Δ0 met15Δ0 ura3Δ0 tma20Δ:KanMX4*	YPD	RiboSeq
8	tma20_rna			YPD	RNA-Seq
9	tma20_sd_ribo			SD	Ribo-Seq
10	tma20_sd_rna			SD	RNA-Seq
11	tma64_ribo	Δtma64	*MATa his3Δ1 leu2Δ0 met15Δ0 ura3Δ0 tma64Δ:KanMX4*	YPD	Ribo-Seq
12	tma64_rna			YPD	RNA-Seq
13	tma64_sd_ribo			SD	Ribo-Seq
14	tma64_sd_rna			SD	RNA-Seq
15	tma20tma64_ribo	Δtma20Δtma64	*MATa his3Δ1 leu2Δ0 met15Δ0 ura3Δ0 tma64Δ:HygMX4 tma20Δ:KanMX4*	YPD	Ribo-Seq
16	tma20tma64_rna			YPD	RNA-Seq
17	tma20tma64_sd_ribo			SD	Ribo-Seq
18	tma20tma64_sd_rna			SD	RNA-Seq

In contrast to a dataset previously obtained in a study by Young et al. [2], here we present ribosome profiling data not only for double knockout yeast strains, but also for strains with individual deletions of *TMA20* and *TMA64*. This allows studying transcriptional and translational changes caused specifically by the absence of individual translation factors TMA20 (MCT-1) or TMA64 (eIF2D). Due to cycloheximide addition to yeast culture medium before harvesting the cells, distribution of mapped reads from Ribo-Seq sets may slightly vary from data described by Young et al. According to a previous study [3], use of the inhibitor in such a fashion is likely to cause blurring of local density effects, but also strengthens Ribo-Seq signals at translation initiation sites, facilitating analysis of ribosome distribution over uORFs.

## 2 Experimental design, materials, and methods

### 2.1 Cell maintenance and cDNA libraries preparation

RNA-Seq and Ribo-Seq сDNA libraries were prepared from total RNA samples or ribosome-bound RNA samples, respectively, for both the wild-type BY4741 yeast strain and three knockout strains (with individually deleted *TMA20* or *TMA64* genes, or with a double deletion of *TMA20* and *TMA64*, hereafter referred as *wt, Δtma20, Δtma64 and ΔΔtma20tma64*, respectively). The libraries were sequenced, resulting in 9 RNA-Seq and 9 Ribo-Seq data sets. Table 1 summarizes information about all of the sequencing experiments.

The experimental procedure in general followed the ribosome profiling protocol described in [1]. Briefly, yeast cells were grown to an exponential phase in either rich YPD (1% yeast extract, 2% peptone, 2% glucose) or minimal SD (0,67% YNB w/o amino acids with ammonium sulfate, 2% glucose, complete amino acid supplementation) media. Cycloheximide was added to yeast media to a final concentration of 100 µg/ml and growth was continued for 3 more minutes; then cells were harvested by filtration, resuspended in polysome lysis buffer (20 mM Tris pH 8.0, 140 mM KCl, 1.5 mM MgCl2, 100 g/ml cycloheximide, 1% Triton), flash frozen in liquid nitrogen and homogenized by grinding. Then a portion of each cell lysate was used for total RNA isolation, while another part was treated with RNase I for polysome disassembly, applied to a sucrose gradient for fractionation, followed by isolation of a monosome fraction and extraction of ribosome-protected mRNA fragments for ribosome profiling. mRNA was isolated using Oligo(dT) beads and ribosome-bound RNA was isolated from sucrose fractions using acidic-phenol extraction. Further ribosome profiling and RNA-Seq library preparations were performed as described previously [1]. Two biological replicates indicated as WT1 and WT2 were performed for wild-type strain maintained in YPD.

### 2.2 Sequence data processing and analysis

Reads were trimmed with cutadapt v1.18 [4] and mapped to the Saccharomyces_cerevisiae.R64-1-1.90 (Ensembl) genome assembly using the respective genome annotation. The read mapping and counting were performed with STAR v2.5.3a [5]. The genomic signal plots (Fig. 1, Fig. 2, Fig. 3) were generated by the svist4get software [6] using bedGraph profiles constructed from bam alignments by samtools and bedtools [7], [8]. Transcription start site coordinates for MAT locus [9] and *GCN4*, *TMA64* and *APC4* genes [10] were used for 5’ UTR mapping in Fig. 2, Fig. 3.

## 3 Data analysis

### 3.1 Ribosome profiling of yeast strains lacking TMA20 and/or TMA64 genes

Translation factor TMA64 and homologs of its N- and C-terminal regions, TMA20 and TMA22 respectively (eIF2D, MCT-1, and DENR in mammals) are proteins involved in translation termination, re-initiation, and ribosome recycling. Initially, eIF2D and heterodimer MCT-1•DENR were assumed to provide a non-canonical translation initiation pathway as they facilitate GTP-independent delivery of Met-tRNA_i_^Met^ and some elongator tRNAs to the 40S ribosomal P-site [11], [12]. In addition, *in vitro* and *in vivo* studies demonstrated that TMA64/eIF2D, TMA20/MCT-1, and TMA22/DENR are able to promote the post-terminational tRNA and mRNA release from the 40S ribosomal subunit both in yeast and mammals [2], [13]. The absence of these factors, together with the 40S recycling failure, led to deregulated translation re-initiation downstream of both short and full-size translated open reading frames in different organisms [2], [14], [15], [16].

The C-terminal regions of TMA64/eIF2D and TMA22/DENR contain the SUI1 domain, which is also present in the translation factor SUI1/eIF1. Structural data indicate that the SUI1 domains of all three factors have similar positions in the P-site of the 40S ribosomal subunit with a conserved β-loop protruding toward a codon-anticodon duplex formed by mRNA and a P-site tRNA [17], [18]. In accordance with biochemical data, this suggests that during recycling, TMA64/eIF2D and the heterodimer TMA20•TMA22 (MCT-1•DENR) may operate in a manner similar to SUI1/eIF1 in translation initiation, or control initiator tRNA access to re-initiating ribosomal complexes after uORF translation.

Raw and analyzed Ribo-Seq and RNA-Seq data sets for wild-type, individual *Δtma20* and *Δtma64,* as well as double *ΔΔtma20tma64* knockout yeast strains were obtained and uploaded into the NCBI Gene Expression Omnibus repository (GEO accession: GSE122039, https://www.ncbi.nlm.nih.gov/geo/query/acc.cgi?acc=GSE122039). The initial analysis revealed that they included deep RNA-Seq and Ribo-Seq data with more than 10 million uniquely mapped and counted reads within gene CDS. In general, in a half of the samples, ~50% of the total library length was composed of uniquely mapped reads. Across all samples, 60 to 90% of them were located within annotated CDS and included in the gene counts. Metagene start- and stop-centric profiles for Ribo-Seq data exhibited clear triplet periodicity (Supplementary Fig. S1). Supplementary Table S1 provides an overview of mapped reads as well as gene-level read counts for all experiments and descriptive statistics on the generated sequencing data.

### 3.2 Examples of data illustrating differential transcription and translation in wt and knockout strains

Two different gene cassettes, MATα and MATa, either of which can be present in the MAT locus of *S.cerevisiae* genome, define the mating type of yeast. Each mating-specific cassette encodes two transcripts directed from the opposite DNA strands by a shared bidirectional promoter: either *MATa1* and *MATa2* in a-type strains, or *MATα1* and *MATα2* in α-type strains (Fig. 1) [9]. All of the transcripts except *MATa2* encode functional proteins – transcription factors that determine mating type or diploid phenotype (reviewed in [19], [20]). While *MATa2* is considered to be non-functional [21], [22], it nevertheless contains two ORFs (Fig. 1), presumably originating from an original coding region with similarity to *MATα2* via an internal frameshift [9], [23]. The second ORF could still encode a remarkably conserved amino acid sequence with a high similarity to a portion of MATα2 [9], [24] that represents its DNA-binding domain [25], [26]. The corresponding protein (MATa2-2) could compete with MATα2 for DNA binding or even have its own transcription factor activity [27]. However, its synthesis should be inhibited by presence of the first ORF in the MATa2 mRNA, which can be regarded as an uORF for the MATa2-2 coding region. Since TMA20 and TMA64 knockout strains have an upregulated translation re-initiation and/or readthrough activities [2], [16], [28], it was interesting to illustrate our ribosome profiling data with a footprint coverage of MATa locus present in BY4741 strain derivatives. As the sequenced S288C strain is MAT*α*, Ribo-Seq and RNA-Seq reads were re-mapped to MATa locus sequence taken from GenBank (accession number V01313.1)[9]. Fig. 1 provides data on Ribo-Seq and RNA-Seq read coverage of MATa locus of the studied yeast strains. This data can be used to explain the role played by TMA20 and TMA64 translation factors in mating and sporulation programs [29], [30], [31], [32].

Another example involves GCN4, the global transcriptional regulator, which is activated during amino acid starvation. Expression of the GCN4 mRNA is controlled by a peculiar mechanism based on differential translation re-initiation on four short uORFs in its 5’ UTR (reviewed in [33]). Fig. 2 shows the 5’ proximal region of the GCN4 transcript, with differential ribosome footprint coverage of uORFs in different strains. Our data can be used for further investigation of TMA20 and TMA64 roles in uORF-mediated translational control of stress response.

*APC4*, the gene encoding a subunit of anaphase-promoting complex, is located in the same genetic locus as *TMA64* and shares a 238-bp promoter region with it. The corresponding mRNAs are synthesized from opposite DNA strands. In the *Δtma64* and *ΔΔtma20tma64* strains the *TMA64* coding sequence was replaced with G-418 or hygromycin resistance gene cassettes (KanMX or HygMX), respectively. Fig. 3 shows differential RNA-Seq coverage of the 238 bp region, flanked by segments of the APC4 and TMA64 coding regions or KanMX/HygMX cassettes, in different yeast strains. This data may likely account for the observed strong genetic interactions of *TMA64* with genes involved in the cell cycle and mitotic checkpoints [34] and cell cycle abnormalities of *TMA64* knockout strains [35].
