# Supplementary material for: Translatome and transcriptome analysis of TMA20 (MCT-1) and TMA64 (eIF2D) knockout yeast strains
Source: Data Brief. 2019 Feb 2;23:103701. doi: 10.1016/j.dib.2019.103701 (PMC6378902; doi:10.1016/j.dib.2019.103701)
Supplement: Supplementary file 1 — Supplementary material [file mmc1.pdf]

Conflict of interest declaration and author agreement form

Title of Paper:
Translatome and transcriptome analysis of TMA20 (MCT-1) and TMA64 (eIF2D) knockout yeast strains

We have no conflict of interest to declare.

This statement is to certify that all Authors have seen and approved the manuscript being submitted. We warrant that the article is the Authors' original work. We warrant that the article has not received prior publication and is not under consideration for publication elsewhere. On behalf of all Co-Authors, the corresponding Author shall bear full responsibility for the submission.

This research has not been submitted for publication nor has it been published in whole or in part elsewhere. We attest to the fact that all Authors listed on the title page have contributed significantly to the work, have read the manuscript, attest to the validity and legitimacy of the data and their interpretation, and agree to its submission to *DATA IN BRIEF*.

All authors agree that author list is correct in its content and order and that no modification to the author list can be made without the written acceptance of all authors and the formal approval of the Editor-in-Chief. All authors accept that the Editor-in-Chief's decisions over acceptance or rejection or in the event of any breach of the Principles of Ethical Publishing in *DATA IN BRIEF* being discovered, of retraction are final.

On behalf of all authors,

Sergey Dmitriev

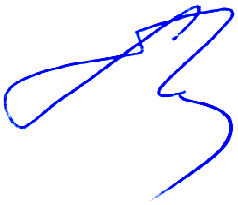

14.12.2018
