# Supplementary material for: Translatome and transcriptome analysis of TMA20 (MCT-1) and TMA64 (eIF2D) knockout yeast strains
Source: Data Brief. 2019 Feb 2;23:103701. doi: 10.1016/j.dib.2019.103701 (PMC6378902; doi:10.1016/j.dib.2019.103701)
Supplement: Supplementary file 3 — Supplementary material [file mmc3.pdf]

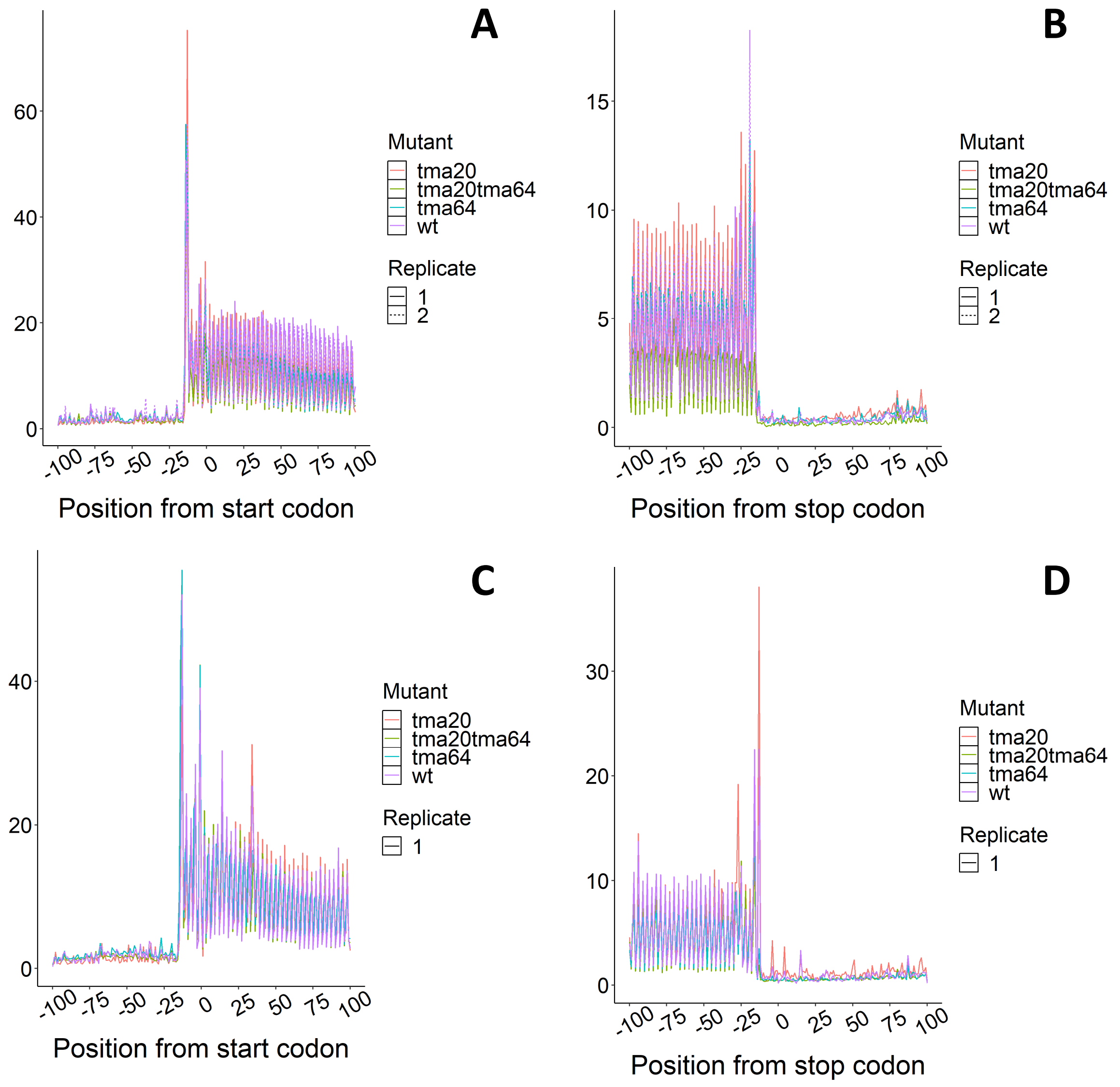

Fig. S1. Metagene profiles demonstrating aggregated coverage with 5’ ends of Ribo-Seq footprints in windows centered at start-codon (A, C) and stop-codon (B, D). Panels A and B: Ribo-Seq data of yeast strains maintained in YPD medium, panels C and D: Ribo-Seq data of yeast strains maintained in SD medium. X axis: relative location; Y axis: the coverage aggregated for all genes; for each gene, the values were normalized to the (total gene coverage / gene length).
